# Metagenomic analysis fecal microbiota of dysentery-like diarrhoea in a pig farm using next-generation sequencing

**DOI:** 10.3389/fvets.2023.1257573

**Published:** 2023-10-17

**Authors:** Xi Chen, Qing Guo, Ying-Ying Li, Tie-Ying Song, Jun-Qing Ge

**Affiliations:** ^1^Institute of Biotechnology, Fujian Academy of Agricultural Sciences, Fuzhou, Fujian, China; ^2^Institute of Animal Husbandry and Veterinary Medicine, Fujian Academy of Agricultural Sciences, Fuzhou, Fujian, China

**Keywords:** dysentery-like diarrhea, metagenomic analysis, fecal microbiota, carbohydrate-active enzyme, antibiotic resistance, virulence factor

## Abstract

Porcine enteric diseases including swine dysentery involves a wide range of possible aetiologies and seriously damages the intestine of pigs of all ages. Metagenomic next-generation sequencing is commonly used in research for detecting and analyzing pathogens. In this study, the feces of pigs from a commercial swine farm with dysentery-like diarrhea was collected and used for microbiota analysis by next-generation sequencing. While *Brachyspira* spp. was not detected in diarrheal pig fecal samples, indicating that the disease was not swine dysentery. The quantity of microbial population was extremely lowered, and the bacterial composition was altered with a reduction in the relative abundance of the probiotics organisms, Firmicutes and Bacteroidetes, with an increase in pathogens like Fusobacterium and Proteobacteria, in which the specific bacteria were identified at species-level. Viral pathogens, porcine circovirus type 2, porcine lymphotropic herpesviruses 1, and porcine mastadenovirus A were also detected at pretty low levels. Carbohydrate-active enzymes (CAZy) analysis indicated that the constitute of Firmicutes and Bacteroidete were also changed. Further, the Kyoto Encyclopedia of Genes and Genomes (KEGG) alignment analysis indicated that the microbiota of diarrheal pigs had a lower ability in utilizing energy sources but were enriched in multi-drug resistance pathways. Comprehensive Antibiotic Resistance Database (CARD) and Virulence Factors of Pathogenic Bacteria (VFDB) analysis indicated that genes for elfamycin and sulfonamide resistance and the iron uptake system were enriched in diarrheal pigs. This revealed potential bacterial infection and can guide antibiotic selection for treating dysentery. Overall, our data suggested that alterations in both the population and functional attributes of microbiota in diarrheal pigs with decreased probiotic and increased pathogenic microorganisms. These results will help elucidate the mechanism of dysentery-like diarrhea and the development of approaches to control the disease.

## 1. Introduction

Porcine enteric diseases, which were associated with various etiological agents (1), could resulting in diarrhea, poor growth performance and variable mortality (2). Swine dysentery is one of the most severe enteric diseases (3), which is often observed in fattening pigs causing significant mucohemorrhagic typhlocolitis accounting for one of the major economic losses for pig producers (4). Although swine dysentery is considered endemic in many regions in the world (5), it can be controlled by consolidated and effective treatments. However, in recent years, some commercial swine farms in Fujian, China, often broken out suspected dysentery-like diarrhea in growing pigs which was extremely difficult to cure.

Although some studies suggested that the swine dysentery infection was associated with the beta-hemolytic strain *Brachyspirahyo dysenteriae* (3), the combinational colonization of several anaerobes was required for the occurrence of swine dysentery (6, 7). Besides, other enteric pathogens, such as *Salmonella enterica* (8, 9), *Lawsonia intracellularis* (10) and *Escherichia coli,* might also cause diarrhea with similar symptoms. Therefore, more than one etiological agent has been suspected to be involved in the disease. Metagenomic sequencing has demonstrated numerous advantages over conventional targeted detection technologies by detecting and characterizing multiple, unexpected, or novel pathogens, and even unculturable microbial species (11–13). Additionally, metagenomic sequencing analysis showed a higher level of phylotype resolution compared to 16S rRNA gene sequencing, thus providing more useful information to discover potential infection pathogens (14), such as Porcine circovirus type 2 (PCV2), Porcine sapelovirus (PSV), Porcine enterovirus, *Escherichia coli*, and *Mycoplasma hyorhinis* (15–19).

In this study, metagenomic analysis was used to characterize fecal microbiota of pigs with suspected dysentery-like diarrhea. The main objective was to explore whether dysentery-like diarrhea was caused by *B. dysenteriae*, and the compositional changes and functional capacity of the microbiota, which can guide antibiotic therapy of the dysentery disease, and strategy for maintaining gut health in swine.

## 2. Materials and methods

### 2.1. Clinical diagnosis and sample collection

Dysentery-like diarrhea was observed in grower-finisher pigs in a commercial swine farm located in Fujian, China, since October 2022. The pigs were administered regular vaccination against Porcine circovirus type 2 (PCV2), Foot and Mouth Disease Virus (FMDV), and other common pathogens. The clinical lesions included a flaccid to fluid-filled colon with serosal hyperemia and variable expansion of the mesocolon by edema. Lesions were multifocal and were frequently observed in the centripetal coils and the apex of the spiral colon but also extended through the centrifugal portion to the terminal aspects over time. Microscopic analysis of smears-prints of the feces did not reveal the parasitic pathogen. Treatment with antibiotics such as mequindox or cefotaxime sodium injection was initiated but resulted in no therapeutic effect. In November 2022, 4 stool samples were collected from both diarrheal pigs (DP group) and healthy pigs (HP group) around 90 days old, respectively. All the sampled pigs were newly diagnosed, and had never received antibiotics or other treatments. The samples were homogenized immediately after collection and stored at −80°C until further processing.

### 2.2. DNA isolation

Genomic DNA was extracted from each stool sample using a Qiamp Fast DNA Stool Mini Kit (Qiagen, Germany) according to the manufacturer’s instructions. The quality of the isolated DNA was assessed by a NanoDrop One instrument (Thermo Fisher Scientific, United States). The isolated DNA was quantified using a Qubit 3.0 benchtop fluorometer (Thermo Fisher Scientific, United States), and stored at −80°C until use.

### 2.3. Library construction and metagenomic sequencing

Sequencing libraries were constructed from isolated DNA samples using an ALFA-SEQ DNA Library Prep Kit (Fangzhou Biological Technology Co, China) according to the manufacturer’s protocols. The library quality was assessed on the Qubit 4.0 fluorometer (Life Technologies, NY) and Qsep400 High-Throughput Nucleic Acid Protein Analysis System (Houze Biological Technology Co, China). At last, the library was sequenced on an Illumina NovaSeq 6,000 platform employing 150 bp paired-end sequencing.

### 2.4. Metagenome assembly and statistical data processing

Raw reads of the sequencing data were converted to FASTQ files using Casava software (Version 1.8.2). Quality assessment was performed with Trimmomactic (Version 0.36) to obtain clean reads. These clean reads were then grouped by subject and assembled using MEGAHIT (Version 1.0.6). After mixed assembly, the Scaftigs were obtained and filtered for statistical analysis. The Scaftigs (≥500 bp) assembled from both single and mixed were used to predict the ORF and filtered by MetaGeneMark (Version 3.38). The CD-HIT (Version 4.7) was employed to remove redundancy and obtain the unique initial gene catalog (Unigenes). The clean data of each sample were then mapped to the initial gene catalog using BBMAP software (Version 38.79) to get the abundance information of individual genes in each sample.

### 2.5. Taxonomy prediction and abundance analysis

DIAMOND software (Version 0.9.30) were used for blast analysis of the Unigenes of bacteria, fungi, archaea, and viruses extracted from the NR database of the NCBI. As each sequence may have multiple aligned results, choose the result of which the e value 1e^−10^ to take the LCA algorithm which is applied to system classification of MEGAN software (Version 6.22.1) to make sure the species annotation information of sequences. The relative abundance and Principal coordinate analysis (PCA) results were based on the abundance table of each taxonomic hierarchy. The microbial composition at each taxonomic level with significant dissimilarities was exhibited by heatmaps on relative abundances and further analyzed by the Wilcoxon rank-sum test to show differences between the organisms. *p* ≤ 0.05 was considered a significant difference.

### 2.6. Functional database annotations

DIAMOND software (Version 0.9.30) was used to perform blast analysis Unigenes with functional databases including the Carbohydrate-active enzymes (CAZy) database, Kyoto Encyclopedia of Genes and Genomes (KEGG) database, Comprehensive Antibiotic Resistance Database (CARD) and Virulence Factors of Pathogenic Bacteria (VFDB) database. The relative abundance of each functional hierarchy was equal to the sum of the relative abundance annotated to that functional level. The gene number table of each sample in each taxonomy hierarchy was obtained based on the function annotation result and gene abundance table. Heatmaps and Linear discriminant analysis Effect Size (LEfSe Version 1.1.01) analysis (the LDA score was 3) were used to find the differences in the microbiome functions between the two groups.

## 3. Results

### 3.1. Changes in unigene frequencies and microbial diversity between diarrheal pigs and healthy pigs

**Figure 1:**
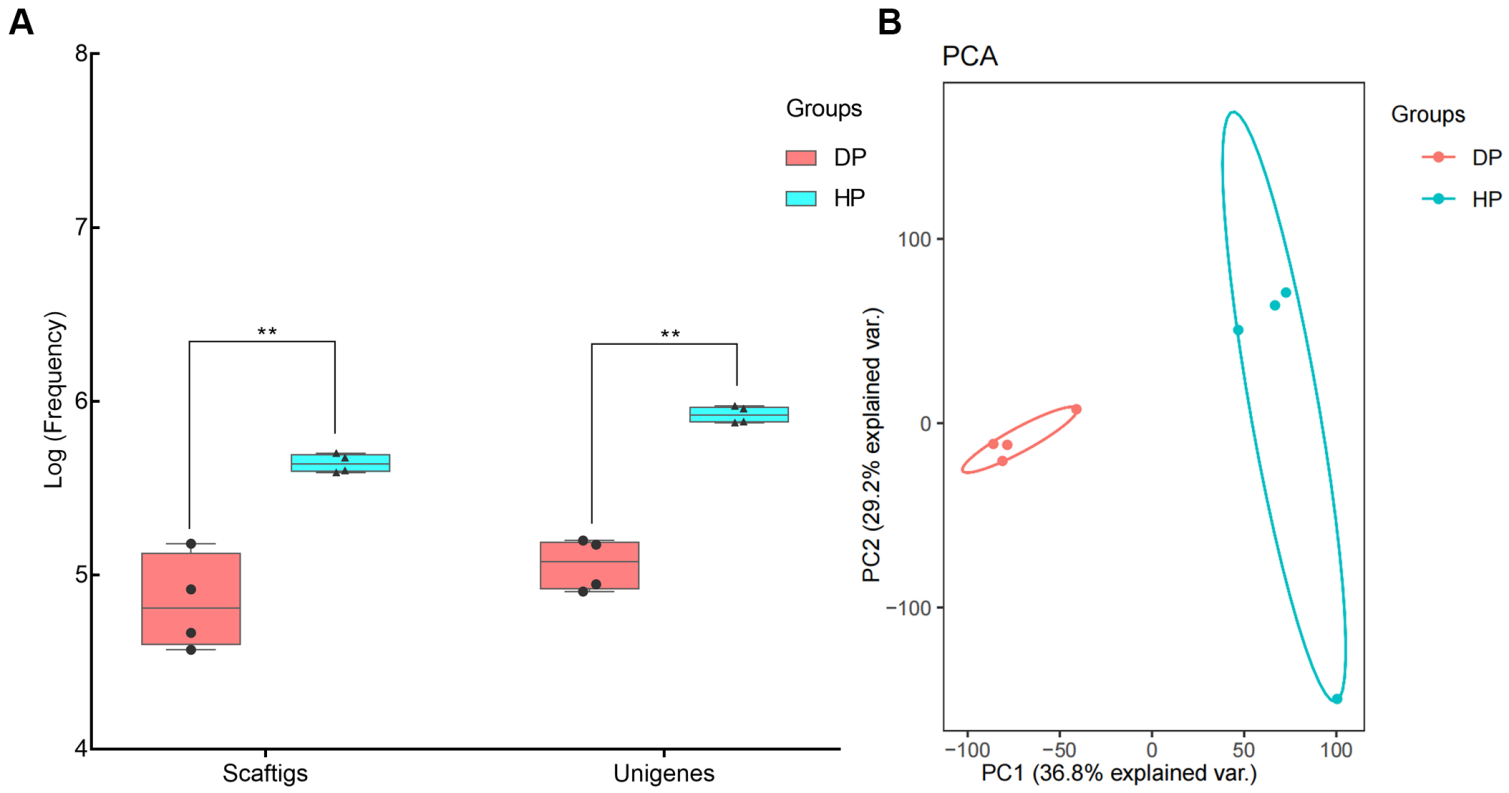
Identification of fecal microbiota of pigs associated with dysentery using metagenomic sequencing. **(A)** Frequency values of Scaftigs and Unigenes identified in the diarrheal and healthy pigs, **(B)** Principal component analysis (PCA) of the fecal microbiota between diarrheal and healthy pigs. DP: diarrheal pigs, HP: healthy pigs, **represented *p*-values <0.01.

Genomic DNA was extracted from stool samples of diarrheal pigs and healthy ones, and used for metagenomic sequencing. An average of 16, 976, 998, and 58, 985, 502 clean reads was obtained from the diarrheal group and healthy group, respectively. Subsequent Blast analysis indicated that 64.5% of the clean reads from the diarrheal group belonged to the host while it was only 3.2% in healthy pigs. An average of 79, 623, and 441, 611 Scaftigs were assembled from the diarrheal group and healthy group, respectively, representing 119, 426, and 840, 750 Unigenes (Figure 1A). The frequencies of Scaftigs and Unigenes had significant differences between diarrheal and healthy pigs. Subsequent Principal component analysis (PCA) verified this difference in the structure of the fecal microbiota between the two groups (Figure 1B).

### 3.2. Bacterial composition comparison between diarrheal and healthy pigs

**Figure 2:**
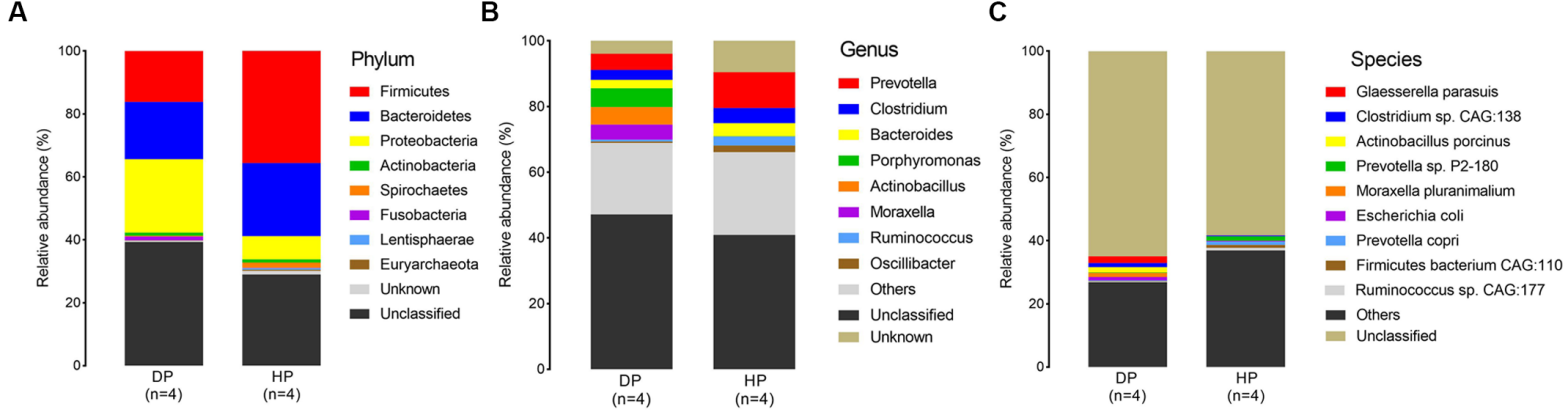
Microbial composition of fecal microbiota in the diarrheal and healthy pigs by metagenomic analysis. **(A)** phylum level, **(B)** genus level, and **(C)** species level. DP: diarrheal pigs, HP: healthy pigs.

Samples from diarrheal and healthy pigs were assessed to evaluate the bacterial composition at the phylum, genus, and species levels. Firmicutes, Bacteroidetes, Proteobacteria, Spirochaetes, and Actinobacteria were the most abundant phyla of the microbiota in both diarrheal and healthy pigs (Figure 2A). However, an increased abundance of bacteria belonging to Firmicutes, Bacteroidetes, and Spirochaetes were found in healthy pigs, while bacteria belonging to Proteobacteria were found in larger abundance in diarrheal pigs. Notably, bacteria belonging to Fusobacteria was observed at an extremely higher level in diarrheal pigs (1.21% on average) than in healthy pigs (0.08% on average) (Figure 2A). Among all identified genera, statistically significant differences were found in the representatives of *Prevotella, Clostridium, Bacteroides, Porphyromonas, Actinobacillus, Moraxella, Ruminococcus,* and *Oscillibacter*. The abundance of *Porphyromonas, Actinobacillus,* and *Moraxella* in the diarrheal group increased to 5.73, 5.37, and 4.57%, respectively, while the abundance of *Porphyromonas, Actinobacillus,* and *Moraxella* in the healthy animals was 0.08, 0.02, and 0.02%, respectively. Further, the relative abundances of *Prevotella, Clostridium, Bacteroides, Ruminococcus,* and *Oscillibacter* were reduced in diarrheal pigs (Figure 2B).

**Figure 3:**
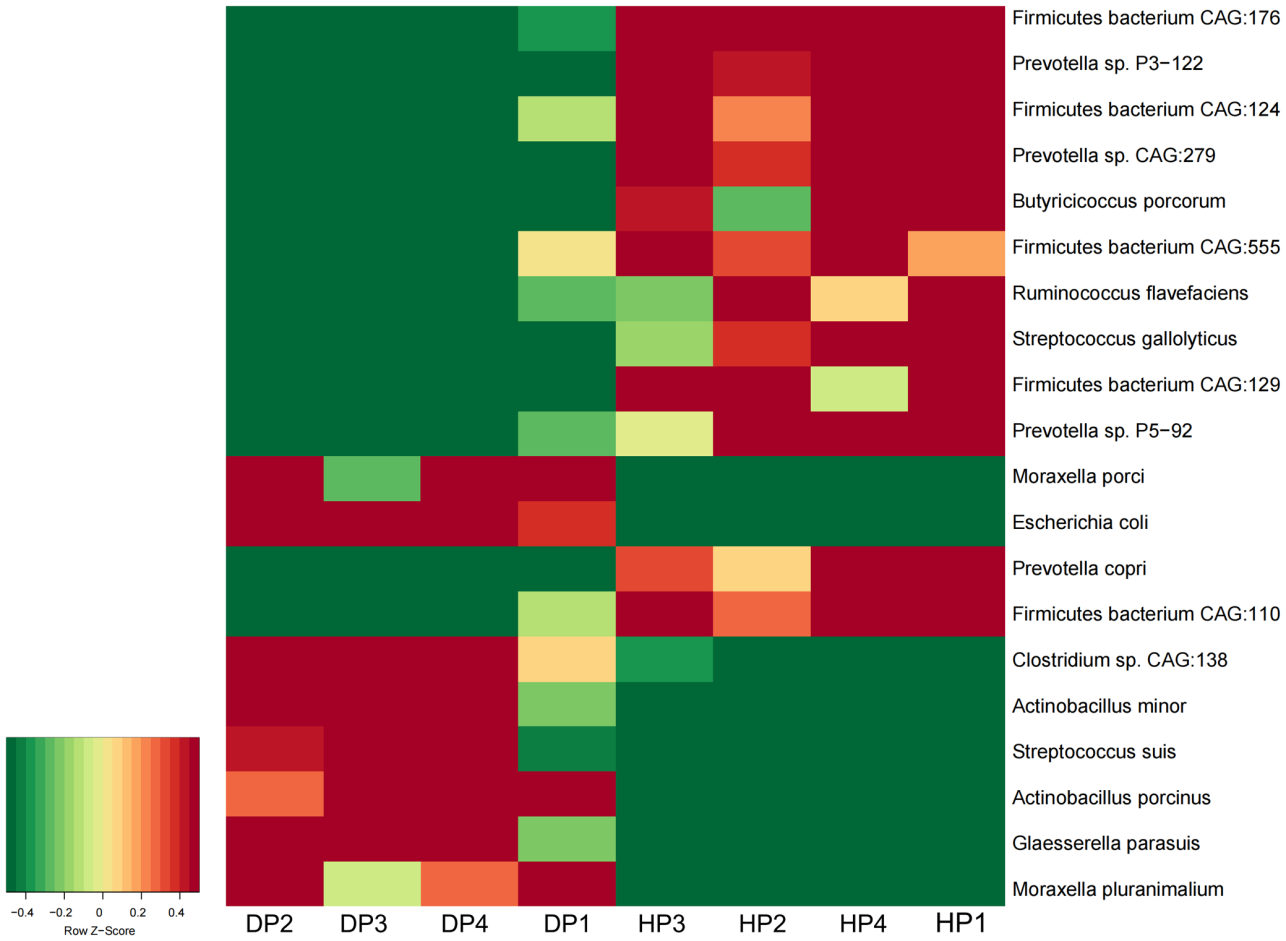
The abundance of the fecal microbiota in diarrheal pigs and healthy pigs analyzed by the Wilcoxon–Rank test. The color scale represents a low expression value in green and a high expression value in red. DP: diarrheal pigs, HP: healthy pigs.

At the species level, *Glaesserella parasuis*, *Clostridium* sp. *CAG:138*, *Actinobacillus porcinus, Moraxella pluranimalium,* and *Escherichia coli* showed an extremely higher abundance in diarrheal pigs, while the abundance of *Prevotella* sp. *P2-180, Prevotella copri, Firmicutes bacterium CAG:110,* and *Ruminococcus* sp. *CAG:177* was lesser in diarrheal pigs (Figure 2C). Subsequent Wilcoxon–Rank test analysis indicated that most of the species enriched in diarrheal pigs were suspected to be pathogenic bacteria, such as those belonging to *Moraxella*, *Actinobacillus* (2 species each), as well as *Clostridium* sp. *CAG:138, Glaesserella parasuis, Escherichia coli*, and *Streptococcus suis.* Furthermore, the species with a higher abundance in the healthy pigs were annotated to be probiotic species, such as 5 species in *Firmicutes,* 4 species in *Prevotella, Ruminococcus flavefaciens*, *Butyricicoccus porcorum*, and *Streptococcus gallolyticus* (Figure 3).

### 3.3. Identification of *Brachyspira hyodysenteriae, Salmonella enterica, Lawsonia intracellularis* and porcine viruses in the diarrheal and healthy pigs

**Figure 4:**
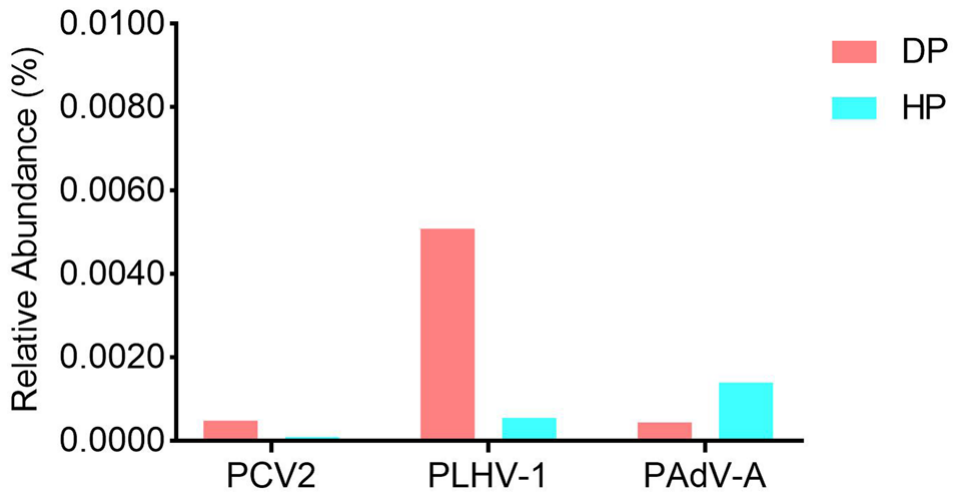
The viral profile analysis of the porcine fecal microbiota in diarrheal and healthy pigs. DP: diarrheal pigs, HP: healthy pigs.

Given that some potential suspected pathogens such as *B. hyodysenteriae, S. enterica, L. intracellularis* and porcine viruses were not identified by the bacterial composition analysis, we performed further independent exploration to examine the presence of these etiologic agents. The results showed that while *B. hyodysenteriae* was not detected, *S. enterica, L. intracellularis,* porcine circovirus type 2 (PCV-2), porcine lymphotropic herpesviruses 1 (PLHV-1), and porcine mastadenovirus A (PAdV-A) were identified in both the diarrheal and healthy pigs. As these bacteria (*S. enterica* and *L. intracellularis*) (data not shown) and the viruses (PCV-2, PLHV-1, and PAdV-A) (Figure 4) were significantly low in abundance with the range of 0.0001–0.005%, and had no difference between diarrheal and healthy pigs, it indicated that these identified microorganism might not be the pathogens causing the dysentery.

### 3.4. Comparison of the functional analysis of the microbiota between diarrheal and healthy pigs

**Figure 5:**
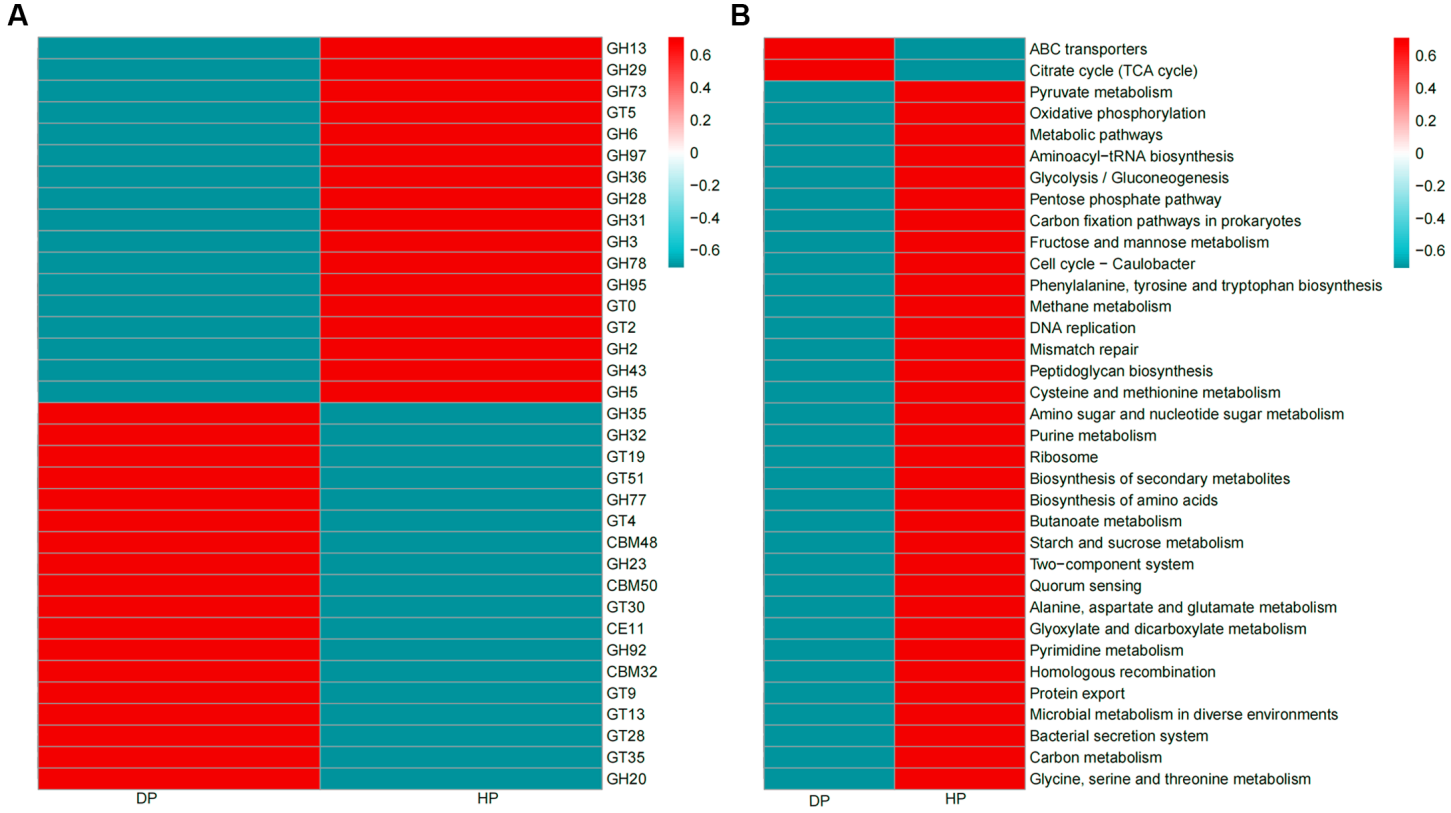
Heatmaps of CAZy enzymes and KEGG pathways of the fecal microbiota of diarrheal and healthy pigs. **(A)** CAZy enzymes, **(B)** KEGG pathways. The color scale represents a low expression value in blue and a high expression value in red. DP: diarrheal pigs, HP: healthy pigs.

CAZy and KEGG analyses were performed to investigate the association of functional capacity of the gut microbiome of pigs with dysentery. 32,852 enzymes were identified by CAZy analysis, among which 19,875 were classified as glycoside hydrolases (GH) and 8,373 belonged to glycosyl transferases (GT). Significant changes were seen in 35 of the most abundant CAZy enzymes. Mainly, 17 enzymes were more abundant in healthy pigs than in diarrheal pigs, most of which belonged to glycoside hydrolases. Of the 18 enzymes with a higher abundance in diarrheal pigs, most of these were classified to be glycosyl transferases (GT4, GT9, GT13, GT19, GT23, GT28, GT35, GT51) and carbohydrate−binding modules (CBM32, CBM48, CBM50) (Figure 5A). Among the identified 35 most abundant KEGG pathways, 33 were enriched in DNA replication, nutrient metabolism, and biosynthesis pathway (e.g., carbon metabolism, starch and sucrose metabolism, glycine, serine, and threonine metabolism, protein export, biosynthesis of amino acids, and biosynthesis of secondary metabolites). These pathways had a higher abundance in the healthy group, while only 2 pathways, ATP-binding cassette (ABC) transporters and citrate (TCA) cycle were enriched in diarrheal pigs (Figure 5B).

**Figure 6:**
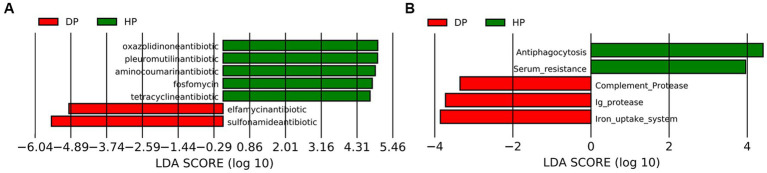
Analysis of the fecal microbiota in diarrheal and healthy pigs by LEfSe analysis. **(A)** CARD database analysis, **(B)** VFDB database analysis. DP: diarrheal pigs, HP: healthy pigs. The X-axis shows LDA scores.

Given that the alterations in the ABC transporters and the TCA cycle might be associated with bacterial multi-drug resistance, further investigation was performed by blast analysis of the Unigenes in CARD and VFDB databases. The CARD analysis identified 30 genes that coded for antibiotic resistance. The changes between the diarrheal group and healthy group were further analyzed by LEfSe. The results demonstrated that five resistance genes towards tetracycline, oxazolidinone, pleuromutilin, fluoroquinolone, and aminocoumarin, were enriched in healthy pigs, while two resistance genes representing sulfonamide and elfamycin, were enriched in diarrheal pigs (Figure 6A). Alignments with VFDB identified 14 gene clusters where significant changes were seen in the abundances of 5 genes. These genes were related to the immunity system (antiphagocytosis, serum resistance, complement protease, and Ig protease) and micronutrient assimilation (iron uptake system). Among these, genes representing complement protease, Ig protease, and the iron uptake system were found to be enriched in diarrheal pigs (Figure 6B).

## 4. Discussion

Porcine enteric diseases like swine dysentery are major problems that cause huge economic losses around the world and are one of the major reasons for the extensive use of antibiotics (2). *B. hyodysenteriae* is regarded as the etiologic agent of swine dysentery, which might appear in the feces of pigs from 1–4 days before the observance of clinical signs (20). These bacteria can be readily identified by culturing on selective media (21). They can also be subjected to PCR amplification with the primers targeting the 16S rRNA gene (22), nox gene (23), or the tlyA hemolysin gene (24) for identification. In this study, no positive results were seen by either of the methods when the collected feces from the diarrheal pigs were analyzed (data now shown), and further metagenomics-based sequencing analysis also did not detect *Brachyspira* spp. Therefore, the disease was not swine dysentery and should be related to another etiologic agents.

Gut microbiota is considered to be a key susceptibility factor for intestinal disorders (25). Previous studies on microbiota also showed that the microbial community diversity could be commonly affected by diarrhea (26). The results of this study also proved that dysentery-like diarrhoea resulted in remarkable alterations in the microbial community, and the abundance of the microbial population was significantly changed by a decline in Unigenes in diarrheal pigs. However, the composition changes on bacteria were quite different from previous metagenomic studies on swine dysentery (27–29). One of the obvious characteristics was the decreasing abundance of the probiotic species, such as Firmicutes and Bacteroidetes. The relative abundance patterns of these two phyla were revealed more distinctively at the genus and species levels. Firmicutes species are constant members of the normal gut microbiota in pigs (30), which can produce short-chain fatty acids, regulate systemic immune responses, maintain energy balance, inhibit opportunistic pathogens, and suppress excessive intestinal inflammation (31). Also different from previous studies (29, 32), *Prevotella*, which accounted for a major part of Bacteroidetes, was observed to be decreased in the diarrheal pigs. As another common probiotic bacterial species, *Prevotella* is frequently detected in the fecal content of pigs (30). It has the ability to degrade plant polysaccharides (33), increase fat accumulation (34), and mediate inflammatory reactions (35) *Prevotella* bacteria also have a negative correlation with the Escherichia group (36). The reduction of Firmicutes and Bacteroidetes might affect the absorption of nutrients and the anti-inflammatory regulation of the host and cause greater damage of diarrhea.

The increase in the abundance of pathogenic Fusobacteria and Proteobacteria in this study is consistent with other studies (28, 37). Both Fusobacterium and Proteobacteria are associated with intestinal inflammatory disorders and are known to play pathogenic roles in the development of pig diarrhoea (38) Specific Proteobacteria species related to dysentery were detected in this study. *M. porci* and *M. pluranimalium* were considered as opportunistic pathogens and potential sources of MCR-Like polymyxin resistance determinants (39). Further, *A. minor* and *A. porcinus* could produce virulent cytotoxins in pigs (40). *E. coli* is a highly significant risk factor for development of diarrhea, and also strongly correlated with gut dysbiosis predisposed to mortality (41). Additionally, *G. parasuis* can trigger a pro-inflammatory response with polyserositis, arthritis, and sepsis in pigs (42). *S. suis* was also shown to induce a pro-inflammatory response with clinical signs such as arthritis, pneumonia, or endocarditis (43). Combining these findings, these species of Proteobacteria might be associated with the dysentery-like diarrhea, but their specific roles in the occurrence of dysentery need further investigation. In addition, although *S. enterica* and *L. intracellularis* are very common intestinal pathogens of swine in China, both of them were detected with pretty low abundance in both diarrheal and healthy pigs, their roles in diarrheal microbiota need further investigation.

Pig gut microbiota could secrete a large repertoire of CAZymes that can breakdown and metabolize polysaccharides (44). These CAZymes are mainly attributed to the probiotic species, Firmicutes and Bacteroidetes (45, 46). Specifically, GH enzymes generally represent the ability of the microbiota to degrade carbohydrates (47), GT and CBM enzymes were likely to be involved in carbohydrate biosynthesis (48, 49). Hence, the down-regulation of GHs and the up-regulation of GTs and CBMs in diarrheal pigs were likely to be attributed to the reduction of the original species and increase in new species of Firmicutes and Bacteroidetes.

The functional capacity of the gut microbiome associated with dysentery was further explored through KEGG analyses. The results showed that the abilities of fecal microbiota to assimilate and utilize multiple energy sources were drastically reduced, which was consistent with the finding that the microbial population was significantly reduced in diarrheal pigs. The fecal microbiota of the diarrheal pigs was enriched in ABC transporter genes and TCA cycle genes. ABC transporters have been considered to play a major role in drug resistance of pathogenic bacteria (50), and the TCA cycle was linked with the number and formation of persister cells, which are phenotypic variants in the bacterial populations (51). These results might be coupled with the increased ability of multi-drug resistance of bacteria in dysentery pigs.

CARD analysis was performed to explore potential antibiotic-resistance genes. A higher abundance of tetracycline, oxazolidinone, pleuromutilin, and fluoroquinolone resistance genes was observed in the microbiota of healthy pigs, which is consistent with previous studies (52, 53). However, elfamycin resistance genes and sulfonamide resistance genes were found to be enriched in diarrheal pigs. Elfamycin is a group of structurally diverse antibiotics commonly produced by Streptomyces strains (54). Sulfonamide resistance is usually harbored by *Enterococcus*, *Bacillus* spp., and *E. coli* (55–57). Thus our results should attract attention to adjusting the application of these antibiotics. It is also a warning about the spreading of these potential pathogens.

Variations in the virulence factors might also help preventing potential pathogens (58). Given a wide range of factors, it is difficult to build a connection between the virulence factors related to the immunity system and the specific pathogens. Further, the significant differences between the two groups could represent the changes in the species of pathogenic bacteria. Additionally, the iron uptake system is the mechanism for some pathogenic bacteria to obtain iron from their hosts (59), which is essential for the growth and successful colonization of these pathogens (60). The species with iron uptake system covered both Gram-positive and Gram-negative bacteria, the pathogens related to ABC transporters in pig microbiota were limited, such as *Campylobacter jejuni*, *Yersinia enterocolitica*, and *E. coli*. Hence these pathogens might also contribute to dysentery development.

## 5. Conclusion

In this study, we confirmed that *Brachyspira* spp. was not the pathogenic cause of local swine diarrhea, although the observed clinical symptoms of the disease were similar to swine dysentery. We found that the fecal microbiota of dysentery pigs was significantly changed, including (i) extremely lowered quantity of the microbial population; (ii) a reduction in probiotic species of Firmicutes and Bacteroidetes, as well as an increase in pathogenic species of Fusobacterium and Proteobacteria; (iii) presence of the PCV-2, PLHV-1, and PAdV-A viral pathogens. Further CAZymes analysis indicated that the structure of Firmicutes and Bacteroidete were also changed. The functional capacity investigation demonstrated that the fecal microbiota of diarrheal pigs had a lower ability to utilize energy sources and had higher multi-drug resistance, which was confirmed by the modifications on antibiotic resistance genes and virulence factor genes. Our results can help in the prevention of the pathogens and selection of suitable antibiotics for treating dysentery-like diarrhea in pigs.

## Data availability statement

The datasets presented in this study can be found in online repositories. The names of the repository/repositories and accession number(s) can be found at: https://www.ncbi.nlm.nih.gov/, PRJNA991276.

## Ethics statement

The animal study was approved by Institute of Biotechnology, Fujian Academy of Agricultural Sciences. The study was conducted in accordance with the local legislation and institutional requirements.

## Author contributions

XC: Conceptualization, Formal analysis, Methodology, Writing – original draft. QG: Conceptualization, Methodology, Writing – review & editing. Y-YL: Methodology, Writing – review & editing. T-YS: Conceptualization, Writing – review & editing. J-QG: Conceptualization, Funding acquisition, Supervision, Writing – review & editing.
